# Neural mechanisms underlying interindividual differences in intergenerational sustainable behavior

**DOI:** 10.1038/s41598-023-44250-z

**Published:** 2023-10-13

**Authors:** Thomas Baumgartner, Emmanuel Guizar Rosales, Daria Knoch

**Affiliations:** 1 Department of Social Neuroscience and Social Psychology, Institute of Psychology, University of Bern, Fabrikstrasse 8, CH-3012 Bern, Switzerland; 2 Translational Imaging Center (TIC), Swiss Institute for Translational and Entrepreneurial Medicine, Bern, Switzerland

**Keywords:** Social neuroscience, Social behaviour

## Abstract

Intergenerational sustainability is a pressing challenge, which is exacerbated by the fact that the current generation must make sacrifices today to ensure the well-being of future generations. There are large interindividual differences in intergenerational sustainable behavior. However, the neural mechanisms underlying these interindividual differences have remained unexplored. Here, we combined fMRI with a consequential intergenerational sustainability paradigm in a sample of 72 healthy students. Specifically, we analyzed task-dependent functional activity and connectivity during intergenerational sustainable decision-making, focusing on the state-like neurophysiological processes giving rise to behavioral heterogeneity in sustainability. We found that differences in neural communication within and between the mentalizing (TPJ/DMPFC) and cognitive control (ACC/DLPFC) network are related to interindividual differences in intergenerational sustainable behavior. Specifically, the stronger the functional connectivity within and between these networks during decision-making, the more individuals behaved intergenerationally sustainably. Corroborated by mediation analyses, these findings suggest that differences in the engagement of perspective-taking and self-control processes underly interindividual differences in intergenerational sustainable behavior. By answering recent calls for leveraging behavioral and neuroscience for sustainability research, we hope to contribute to interdisciplinary efforts to advance the understanding of interindividual differences in intergenerational sustainability.

## Introduction

Intergenerational sustainability, commonly defined as meeting the needs of the present generation without compromising the ability of future generations to meet their own needs, poses pressing challenges for societies and humanity^1,2^. These challenges include, for example, long-term investments in infrastructure, education, and renewable energy, the reduction of public debt, the provision and maintenance of social insurance systems, and global climate change. Despite widespread awareness and concern regarding these challenges, there is considerable variation in the extent to which individuals behave sustainably^3–5^. What mental processes and neural mechanisms drive these interindividual differences in intergenerational behavior, however, has largely remained an open question.

Scholars have increasingly begun to advocate for the integration of neuroscience into sustainability research, as it holds great promise for unlocking a deeper understanding of the complex mental processes and neural mechanisms underlying sustainable decision-making that might not be obtained by behavioral observations or self-reports alone^6–10^. Specifically, neuroscientific methods can provide objective measures that are free from response biases and demand characteristics and allow investigating and distinguishing different decision-making processes driving behavior that are difficult to access by introspection alone. In a recent study, we leveraged neuroscience for examining interindividual differences in intergenerational sustainable behavior^11^. We found that task-independent (offline) neuroanatomical differences in cortical thickness could act as trait-like neural markers illuminating the sources of behavioral heterogeneity in intergenerational sustainability. Here, we used task-dependent (online) neurophysiological measures of the decision processes during intergenerational sustainable decision-making to identify more proximal mechanisms that give rise to differences in intergenerational sustainability. Specifically, we used fMRI to investigate whether differential patterns of task-dependent functional brain activity and connectivity could explain interindividual differences in intergenerational sustainable behavior.

The presence of great interindividual variability in sustainable behavior should come as no surprise, considering that intergenerational sustainability poses complex, unique, and challenging dilemmas. These dilemmas force people of the present generation to choose between sacrificing their own benefits to provide benefits for future generations or maximizing their own benefits at the expense of future generations. Importantly, members of the present generation *unidirectionally* impact future generations and do not directly benefit or suffer from the delayed consequences of their own actions. This clearly sets intergenerational sustainability dilemmas apart from social dilemmas that occur within a single generation^12,13^.

Previous work suggests that the challenging nature of intergenerational sustainability dilemmas is rooted in the unique combination of the social and temporal distance between the present and future generations^13,14^. These psychological distances are related to preferential biases in single-generation contexts. In these contexts, people tend to prioritize the benefits of themselves or socially close others rather than socially distant others^15^, which is known to drive intergroup bias in single-generation social dilemmas^16^. Moreover, people tend to prefer immediate smaller benefits over later larger benefits, which constitutes temporal discounting in single-generation intertemporal choice tasks^17^. Yet, the unique interplay of social and temporal dimensions renders intergenerational sustainability dilemmas distinctively more complex than dilemmas only involving a single generation. For example, in sharp contrast to single-generation intertemporal choice tasks, those who forgo immediate benefits (i.e., the present generation) are not the ones who reap the long-term benefits of their behavior (i.e., future generations) in intergenerational sustainability dilemmas. Thus, the investigation of mental processes and neural mechanisms driving interindividual differences in intergenerational sustainable decision-making might be informed by, but cannot be reduced to, research on single-generation dilemmas. To assess and investigate intergenerational sustainability, research needs to model its unique complexity.

Here we assessed behavior and neural responses during intergenerational sustainable decision-making in a well-controlled laboratory setting. For this purpose, we combined fMRI with an intergenerational sustainability dilemma game, modelling the key features and contingencies of intergenerational behavior, i.e., the social and temporal distance between benefactors and beneficiaries, the temporal delay between actions and consequences, the unidirectionality of interactions, and the absence of reciprocity. In this game, four participants partaking on the same day formed the present generation, whereas four other participants partaking one week later formed the (future) next generation. Participants did not know each other neither within nor across generations. Over several independent trials, each participant could extract points from a common pool shared with the other participants of the present generation. Crucially, overharvesting of the pool either negatively affected the next or the present generation. In trials affecting the next generation (*Gen*_*next*_), the payoff of every participant of the next generation was reduced considerably if the present generation collectively overharvested the pool. In trials affecting the present generation (*Gen*_*pres*_), overharvesting reduced the payoff of every participant of the present generation. The difference in extracted points in *Gen*_*next*_ minus *Gen*_*pres*_ served as a measure of interindividual differences in intergenerational sustainable behavior that we labelled extraction score. In this extraction score, values close to zero represent more intergenerational sustainable behavior while higher positive values indicate more intergenerational *un*sustainable behavior. Thus, this setup allowed us to model the characteristic features and contingencies of real intergenerational sustainable behavior: Participants had to cooperate with anonymous others of the present generation by incurring real costs to benefit other unknown participants of future generations rather than themselves. Additionally, participants of future generations would experience the consequences of present generation participants’ behavior with temporal delay and could not reciprocate (or retaliate).

Given the sparse literature on interindividual differences in intergenerational sustainability, it is difficult to derive clear predictions regarding neural mechanisms driving these differences. Therefore, hypotheses needed to be based on findings concerning related single-generation social dilemmas, intergroup situations, and intertemporal choice. Even though intergenerational sustainability cannot be reduced to these phenomena, they nevertheless might inform tentative hypotheses.

Individuals who engage more in taking the perspective of future others may be better able to cooperate to benefit future generations by bridging the social distance from them. For example, perspective-taking with others is related to cooperation in social dilemmas^18,19^, reduces intergroup bias^20^, and increases sustainable^21,22^ and pro-environmental behavior^23^. Neuronally, perspective-taking processes are mainly supported by functional activity in and connectivity between central nodes of the mentalizing network, specifically the dorsomedial prefrontal cortex (DMPFC) and the temporoparietal junction (TPJ)^24–29^. For instance, reduced activity in and connectivity between the DMPFC and TPJ were associated with stronger punishment of norm violating outgroup (vs. ingroup) members in a third-party punishment paradigm^30^, suggesting that a lack of perspective-taking with outgroup members might drive intergroup bias in these single-generation intergroup situations. Thus, we expected that individuals with greater activity in and connectivity between the TPJ and DMPFC during making decisions affecting the next (vs. present) generation would behave more sustainably.

Differences in the processing of cognitive conflicts and the subsequent employment of self-control might be another driver of interindividual variability in intergenerational sustainable behavior. Self-control allows individuals to advance one goal over another goal when these are perceived to be in conflict^31^. Such cognitive conflicts arise when mutually exclusive goals are simultaneously activated^32^, as for example when individuals are forced to decide between serving self-interests vs. collective interest, benefitting the ingroup at the expense of the outgroup, or receiving sooner smaller vs. larger later rewards. Thus, cognitive conflicts and their regulation by self-control processes are inherent and common to single-generation social dilemmas, intergroup, and intertemporal choice situations alike. At the neural level, such conflicts and their resolution via self-control are processed in a cognitive control network including the anterior cingulate cortex (ACC) and the dorsolateral prefrontal cortex (DLPFC)^32–34^. Regarding the specific functions of these brain regions, evidence suggests that the ACC monitors conflicts based on input it receives from other brain areas and specifies whether and to which extent cognitive control needs to be employed. The ACC then outputs this information to the DLPFC, which implements cognitive control accordingly. In single-generation phenomena related to intergenerational sustainability, greater activity within the cognitive control network during decision-making in social dilemmas, intergroup situations, and intertemporal choice has been associated with more pro-social, less biased and more patient decisions^35–40^, whereas disrupting the cognitive control network by inhibitory brain stimulation was found to reduce these decisions^41–43^. Based on these findings, we therefore hypothesized that individuals with greater functional activity and connectivity within the cognitive control network and between the mentalizing and the cognitive control network when making decisions affecting the next (vs. present) generation would behave more sustainably.

## Methods

### Participants

We assessed behavioral and brain data of 77 healthy students of the University of Bern. Five participants were excluded because they failed the comprehension check (see game-specific questions). No participant had to be excluded because of movement artifacts, anomalies in the brain data or technical problems. The final sample consisted of 72 participants (40 female, 32 male, mean age ± SD = 21.75 ± 2.71). The study was conducted according to the principles expressed in the Declaration of Helsinki and was approved by the ethics committee of the canton of Bern (no. 2020–00302). Participants signed informed written consent prior to the participation in the study.

### Procedure

The present study is part of a larger project investigating brain anatomical and functional correlates of interindividual differences in intergenerational sustainable decision-making and behavior. Here, we focused on task-dependent functional brain activity and connectivity to shed light on the mental processes and neural mechanisms driving interindividual differences in intergenerational sustainable decision-making. Results of a neural trait approach study investigating the anatomical sources of interindividual differences in intergenerational sustainable behavior are reported elsewhere^11^. Please take note that both the structural and functional studies were conducted in parallel, operating independently from one another. The conclusions drawn from the structural study did not inform the hypotheses or analyses undertaken in the functional study.

Participants showed up for one session in which their behavioral and brain data was acquired. Outside of the scanner, participants first read the instructions for the intergenerational sustainability dilemma game, which they then played inside the scanner while undergoing functional MRI. Directly after game completion, participants answered game-specific questions and the comprehension check. Next, participants’ structural MRI was acquired. At the end of the session, participants received their fixed show-up fee of CHF 40. Two weeks after the session, participants received a link to an online questionnaire (see assessment of subjective values) and their additional payment, which depended on their own and others’ behavior (see below).

### Intergenerational sustainability dilemma game

We aimed at assessing intergenerational sustainable decision-making and behavior independently of any specific sustainability context as, e.g., environmental or climate behavior. Accordingly, we designed a game inspired by the seminal study of Hauser and colleagues^44^, focusing on the consequences that the behavior of the present generation entails for a future generation. Please note that the same paradigm was used and described in our previous study^11^. To model the succession of different generations separated by a temporal delay, four participants who completed their session on the same day formed the present generation, while four other participants who would partake about 7 days later constituted the next generation. Like in the seminal work by Hauser and colleagues^44^ and ensuing studies^45–49^, participants were explained in the instructions that the game included a sequence of “groups” separated by a temporal gap and that participants’ behaviors affected the payoff of the “next group” or “present group”. Because the game modeled key features of intergenerational sustainability (social and temporal distance between benefactors and beneficiaries, temporal delay between actions and consequences, unidirectionality, and lack of reciprocity, see Introduction section for details), the “next group” can be considered as a proxy for a future generation^21,44–49^. By truthfully and transparently communicating all rules of the game to participants, we followed standard principles for the application of behavioral economic games.

In each of 16 independent trials, participants could extract between 0 and 20 points (in increments of 2 points) from a pool shared with the other three participants of the present generation. Each point was worth 1 CHF. Extraction of points took place under two conditions, which were equally distributed over the 16 trials in a pseudo-randomized order. In eight trials, participants of the present generation were informed that if they collectively extracted more than 40 points, every participant’s payoff of the present generation was reduced by 80% for that trial. In the other eight trials, participants were informed that if the present generation’s collective extraction exceeded 40 points, the payoff of every participant of the next generation would be reduced by 80% in that trial, while the payoffs of the participants of the present generation were not affected. Thus, trials differed in whether the present or the next generation’s payoff was affected if the present generation exceed the threshold of 40 points. Accordingly, we named the experimentally manipulated factor Affected Generation (*Gen*) and refer to its two conditions as *Gen*_*pres*_ for trials affecting the present generation and as *Gen*_*next*_ for trials affecting the next generation. Note that *Gen*_*pres*_ trials served as within-subject control condition for behavioral and brain measures in *Gen*_*next*_ trials, which were the main focus of interest. Accordingly, behavioral and brain measures in *Gen*_*next*_ trials were analyzed relative to corresponding measures in *Gen*_*pres*_ trials using difference values (*Gen*_*next*_–*Gen*_*pres*_, for example see extraction score below).

Each trial started with a fixation baseline epoch lasting for 9–13 s (randomly jittered). Then, participants were presented with the decision screen. In the top half of the screen, they saw which generation (present vs. next) would be affected by the present generation’s decision (word frame: “affected group: present” or “affected group: next”) and they were reminded of the collective extraction threshold of 40 points and of the payoff reduction consequences if the threshold was exceeded. After a delay of 5 s, the bottom half of the decision screen was revealed, in which participants saw the question “How many points would you like to extract?”. To give their answer, participants were presented with a horizontal scale ranging from 0 to 20 (in increments of 2) beneath the question. Participants could input their decision by using two navigation buttons and a confirmation button. Navigation consisted of moving a red dot indicating the currently selected option (starting point of the scale was chosen randomly in each trial) to the desired scale point. A trial ended when participants confirmed their decision (within a time restriction of 13 s starting from the onset of the trial). The intergenerational sustainability dilemma game was programmed in z-tree, which is a software for social interaction experiments^50^.

After all participants of the present generation had completed their sessions, two trials were selected randomly to be paid out (accounting for potential payoff reductions). Participants were then given feedback on their generation’s collective behavior and its consequences for the present and next generation. Two weeks later, participants received the variable amount they earned in the game (in addition to the fixed show-up-fee of CHF 40, which they had received directly after their session).

### Behavioral extraction score and behavioral types

**Figure 1:**
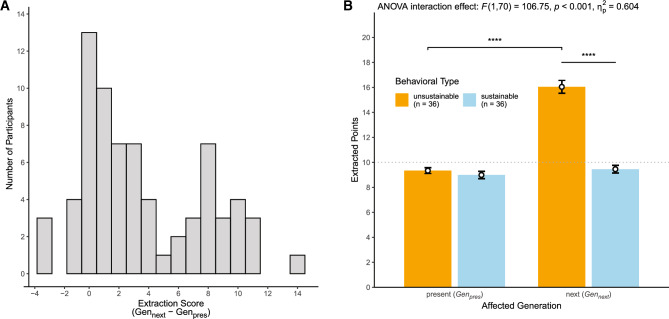
Histogram of the extraction score and categorization of distinct behavioral types. The histogram in panel (**A**) shows considerable interindividual variability in the extraction score, which we used to index interindividual differences in sustainable behavior based on participants’ behavior in the intergenerational sustainability dilemma game (see Methods section). Higher positive values on this score indicated that a participant on average extracted more points in *Gen*_*next*_ compared to *Gen*_*pres*_ trials, whereas lower values around zero indexed that a participant extracted an equal number of points in both conditions. To better understand the difference values in the extraction score, we identified two behavioral types by categorizing participants as sustainable if their median extraction in *Gen*_*next*_ trials was ≤ 10 points and as unsustainable otherwise (see Methods section). The bar graph in panel (**B**) illustrates that unsustainable (vs. sustainable) participants extracted considerably more points in *Gen*_*next*_ trials, whereas the two types did not differ in *Gen*_*pres*_ trials. Thus, unsustainable participant’s higher extraction scores reflect more unsustainable behavior toward the next generation, whereas sustainable participants lower extraction scores around zero index more sustainable behavior. The dotted line represents the 10 points that each participant of the present generation could extract on average without reducing payoffs for the present (in *Gen*_*pres*_ trials) or next (in *Gen*_*next*_ trials) generation. Error bars depict standard errors of the means and asterisks denote significant differences (**** *p* < 0.001) based on dependent and independent t-tests. For completeness, we also report the result of the significant two-way mixed ANOVA interaction effect.

To index interindividual differences in intergenerational sustainable behavior, we created an extraction score (see Fig. 1A) for each participant by calculating the difference between the mean of extracted points in trials affecting the next generation minus the mean of extracted points in trials affecting the present generation (*Gen*_*next*_–*Gen*_*pres*_). Thus, higher positive values indicated that participants extracted more points if the next compared to the present generation was affected by their decisions. That is, higher positive values indexed more *un*sustainable behavior towards the next generation (see behavioral results). Lower values around zero indicated that participants extracted an equal number of points if the next or the present generation was affected. That is, lower values indexed more sustainable behavior towards the next generation (see behavioral results). Negative values indicated that participants extracted less points if the next compared to the present generation was affected. Note that this extraction score served as the main behavioral variable of interest in all subsequent analyses.

To disentangle and better understand the difference values in the extraction score, we categorized participants into behavioral sustainability types by following the same rationale as described in our previous study^11^. In trials affecting the next generation, each of the four participants of the present generation could extract up to 10 points without risking to collectively extract more than 40 points, which would reduce the payoff of the next generation. Thus, 10 points represent a reasonable individual sustainability threshold. Therefore, we categorized participants as sustainable if their median extraction in trials affecting the next generation was lower or equal to 10 points and as unsustainable otherwise. Please note that this procedure is not the same as a median split but a categorization based on the a priori defined sustainability threshold. As visualized in Fig. 1B, this approach allowed us to break down the extraction score by Behavioral Type (unsustainable vs. sustainable) and Affected Generation (*Gen*_*pres*_ vs. *Gen*_*pres*_), which facilitated understanding these difference values and their associations. Note, however, that all primary statistical inferences are derived from regression analyses using the full variance of the extraction score. If additional statistical inferences based on the behavioral types are reported, these are of secondary nature and are only intended to ease the understanding of regression findings.

### Game-specific questions

Our hypotheses focused on perspective-taking and self-control as potential drivers of interindividual differences in intergenerational sustainable behavior. To assess whether participants indeed were engaged in these socio-cognitive processes during the intergenerational sustainability game, we formulated game-specific questions tapping into perspective-taking and self-control. The same questions were used and described in our previous study^11^. We asked participants to indicate their agreement to specific statements on a scale from 1 (“do not agree at all”) to 11 (“completely agree”) directly after game completion. In a first block, participants were presented with the statements that, in *Gen*_*next*_ trials, (1) putting themselves in the shoes of members of the next generation and (2) putting themselves in the shoes of members of the present generation influenced their decision. These two statements aimed at assessing participants’ engagement in perspective-taking processes during *Gen*_*next*_ trials. Additionally, participants indicated their agreement to the statement that, in *Gen*_*next*_ trials, (1) they were tempted to extract more than 10 points and that (2) they tried to resist this temptation. These two statements were designed to reflect that behavior resulting from (failed) self-control processes involves (1) a desire conflicting with a higher-order goal (a temptation) and (2) a (lack of) effort to control this desire (effort to resist the temptation)^51,52^. In a second block, participants answered the same statements as in the first block but now concerning *Gen*_*pres*_ trials. However, they were not asked to indicate the extent to which they took the perspective of members of the next generation because this statement was not sensible in *Gen*_*pres*_ trials.

We calculated participants’ differential engagement in perspective-taking in *Gen*_*next*_ trials by taking the difference between perspective-taking with members of the next generation (Target of Perspective-Taking: *TP*_*next*_) minus perspective-taking with members of the present generation (*TP*_*pres*_). To quantify participants’ differential engagement in self-control, we calculated two difference scores. We (1) took the difference between participants’ temptation in *Gen*_*next*_ minus *Gen*_*pres*_ trials (Δ temptation) and we (2) took the difference between participants’ effort to resist the temptation in *Gen*_*next*_ minus *Gen*_*pres*_ trials (Δ resist_temptation_). In statistical analyses (see mediation results), we focused on Δ resist_temptation_ while entering Δ temptation as a covariate. Because we statistically adjusted for Δ temptation, we considered effects regarding Δ resist_temptation_ as representing effects concerning differential engagement in self-control^51,52^.

As a comprehension check, participants had to indicate the average number of points each member of the present generation could extract without reducing the payoff of the next generation. Hence, this question allowed us to ensure that participants did not accidentally behave unsustainably while believing they were behaving sustainably (leading to the exclusion of five participants, see Participants section).

### Assessment of subjective values

Participants answered the Schwartz Values Scale (SVS)^53^ online after the experimental session. They rated how important 16 values were for them as leading principles in life (1: “not at all important”, 6: “very important”). The SVS is divided into four subscales, which assess altruistic, egoistic, hedonic, and biospheric values. These values have been argued to play crucial roles in pro-environmental^53^ and intergenerational behavior^13^. Thus, we tested whether interindividual differences in intergenerational sustainable behavior were associated with differences in these values by regressing the extraction score on the four SVS subscales in a multiple regression.

### Functional and structural data acquisition

All MRI data were acquired on a Siemens MAGNETOM Prisma 3 Tesla whole-body scanner using a 64-channel head coil. The functional session started off with a localizer scan followed by the intergenerational sustainability game. The game was projected onto a screen that the subjects viewed through an angled mirror mounted to the head coil. Subjects made their responses on a three-button response box in their right hand. While subjects were playing the game, we acquired gradient echo T2*-weighted echoplanar images (EPIs) with blood oxygenation level dependent (BOLD) contrast (308 volumes per subject, 54 slices per volume, multi-slice mode: interleaved, acceleration factor slice: 3 (parallel acquisition of three slices), field of view: 235 × 235 × 135 mm, slice thickness: 2.5 mm, no gap, repetition time: 1300 ms, echo time: 30 ms, flip angle 80°). Volumes were acquired in axial orientation at a 15° tilt to the anterior commissure–posterior commissure line. After the functional session, T1-weighted 3D-modified driven equilibrium Fourier transformation (MDEFT) images were acquired from each subject (176 slices, field of view: 256 × 256 × 176 mm, slice thickness: 1 mm, no gap, repetition time: 7.93 ms, echo time: 2.49 ms, flip angle: 16°).

### Preprocessing of functional and structural data

The statistical parametric mapping software (SPM12, Version v7771, https://www.fil.ion.ucl.ac.uk/spm/software/spm12/) implemented in Matlab (Version R2020b) was used for preprocessing and statistical analyses. All functional images were realigned and unwarped to the mean EPI image to correct for movement artefacts and susceptibility distortions. We then corrected for time of slice acquisition within a repetition time (reference time: 640 ms), taking into account the parallel acquisition sequence. Realigned and slice time corrected images were then normalized to stereotactic space (3 × 3 × 3 mm^3^) corresponding to the Montreal Neurological Institute (MNI) with parameters estimated from the co-registered structural image using the segmentation algorithm. Finally, images were smoothed using an 8-mm full-width-at-half-maximum Gaussian kernel.

### fMRI Analyses: functional activity

The statistical analyses of the fMRI data were also performed in SPM12. We modeled each participants’ BOLD response with a general linear model (GLM) that was estimated using a standard hemodynamic response function and a high-pass filter of 128 Hz, as well as a correction for intrinsic autocorrelations. To increase signal to noise ratio, we minimized global intensity changes by scaling each image to the grand mean. The GLM contained two regressors of interest as boxcar functions: (1) decisions in trials affecting the next generation (*Gen*_*next*_) and (2) decisions in trials affecting the present generation (*Gen*_*pres*_). Each of these decisions were modeled with durations from the respective onset of the decision screens until the decisions were individually confirmed via button press (mean ± SD: response time in *Gen*_*next*_: 8.67 ± 1.13 s, response time in *Gen*_*pres*_: 8.56 ± 1.21 s, no significant differences between the two conditions: *p* = 0.122). Note that a first-level model with additional regressors separately modeling the motor response led to highly similar and statistically undistinguishable findings for the decision phase (which in this case was modelled until the first button press). Please also note that a first-level model with a parametric modulator of extraction behavior is not feasible due to high consistency in the subjects’ decisions (i.e., small within-subject variability). As nuisance regressors, the six motion parameters produced during realignment were included as additional regressors in the SPM analysis to account for residual effects of scan-to-scan motion.

Based on these first-level GLM estimates, we computed the following single-subject contrasts of regression coefficients (beta estimates) for the decision phase: *Gen*_*next*_ > *Gen*_*pres*_. These first-level contrasts were then taken to second-level random effects analyses, in which we regressed the activation differences between the two conditions (*Gen*_*next*_ > *Gen*_*pres*_) on the interindividual differences in extraction score (*Gen*_*next*_–*Gen*_*pres*_), i.e., we tested whether differences in functional activity were associated with interindividual differences in intergenerational sustainable behavior.

### fMRI analyses: functional connectivity

Task-dependent and seed-based functional connectivity was analyzed with the SPM-based connectivity toolbox CONN (version 20b)^54,55^. For this purpose, first-level SPM design matrices of each subject (as previously described) and the fully preprocessed functional and anatomical images (including normalized gray matter, white matter and CSF images derived from the segmentation process) were first imported in the CONN toolbox. The seed areas used for functional connectivity analyses were defined as a 5 mm sphere around the peaks identified in other analyses (see Results section for details). Furthermore, an additional denoising process^56^ recommended for functional connectivity analysis was applied to eliminate confounds of white matter, CSF, subject motion and effects of the task (*Gen*_*pres*_, *Gen*_*next*_, and resting baseline). This procedure allows to remove the temporal timeseries of each confound from functional images and to apply a band-pass filter (0.01 Hz < *f* < 0.10 Hz) to the residual timeseries. Adding regressors accounting for task effects in this preprocessing step allowed preventing that the main effects of the task drove the estimation of the functional connectivity. First-level functional connectivity for each subject and condition (*Gen*_*pres*_, *Gen*_*next*_) was computed using weighted bivariate regression coefficients between the seed timeseries (left TPJ and dorsal ACC, see Results section for details) and timeseries from all other voxels in the brain. These first-level condition-specific connectivity estimates were then taken to second-level random effects analyses, in which we regressed the functional connectivity differences between the two conditions (*Gen*_*next*_ > *Gen*_*pres*_) on the interindividual differences in the extraction score (*Gen*_*next*_–*Gen*_*pres*_), i.e., we tested whether differences in functional connectivity between the seed areas (left TPJ / dorsal ACC) and other areas/voxels in the brain were associated with interindividual differences in intergenerational sustainable behavior.

### fMRI analyses: statistical inferences

We looked for functional activity and connectivity effects as a function of intergenerational sustainable behavior across the whole brain as well as across a priori defined regions of interest (see Introduction section) involved in perspective-taking (DMPFC and TPJ) and self-control (ACC and DLPFC). For this purpose, we created a mask consisting of the bilateral ACC, bilateral middle frontal gyrus (MFG, which the DLPFC is part of), the DMPFC and bilateral TPJ. For the definition of the MFG and ACC, we used the automated anatomical labelling atlas as provided in the WFU Pickatlas toolbox^57^. For the definition of the DMPFC and TPJ, we used a meta-analysis on social cognition^29^ to define peaks in the left TPJ (x = ‑49, y = ‑58, z = 22), the right TPJ (x = 53, y = ‑54, z = 22), and the DMPFC (x = ‑3, y = 48, z = 30), which consisted of the average coordinates of areas that had consistently been found activated in perspective-taking tasks (including goal, intention, and trait inferences as well as morality judgments). Then, we created 20 mm spheres centered on these coordinates by using the WFU Pickatlas toolbox.

We considered findings significant if they survived false discovery rate (FDR) correction on peak- or cluster-level at *p* < 0.05 across the whole brain (whole-brain FDR-corrected) or across the small volume mask defined above (small-volume FDR-corrected). For cluster inference, we used a cluster-defining threshold of *p* < 0.001. For significant activity and connectivity findings (based on the calculated regression analyses, see above), we extracted the contrast estimates (*Gen*_*next*_ > *Gen*_*pres*_) of the corresponding clusters (thresholded at *p* < 0.001 or *p* < 0.005) and used these contrast estimates for creating scatter plots as well as for mediation analyses (see below). Furthermore, we also extracted the activity and connectivity estimates in the significant clusters (again thresholded at *p* < 0.001 or 0.005) separately for the two conditions (*Gen*_*next*_, *Gen*_*pres*_) and plotted these values in bar plots, broken down for the two behavioral types (unsustainable vs. sustainable, see above). These post-hoc visualizations (and corresponding analyses, see Results section) using the disentangled activity and connectivity values (and not the contrast estimates representing difference values) helped to better understand what drives the observed effects in the regression analyses. However, we would like to emphasize that the primary statistical inferences are derived from the regression analyses.

### Statistical analyses of behavioral and extracted functional data

For primary analyses, we conducted linear regression analyses and reported Pearson correlation coefficients *r* and *r*^2^ for effect sizes by using R’s stats package’s functions lm() and cor.test(). For secondary analyses, we conducted two-way mixed between-within-subjects analyses of variance (ANOVAs) in R by using the package afex (version 1.2–1). We used Behavioral Type (unsustainable vs. sustainable) as between-subject factor and Affected Generation (*Gen*_*pres*_ vs. *Gen*_*next*_) or Target of Perspective-Taking in *Gen*_*next*_ (*TP*_*pres*_ vs. *TP*_*next*_) as within-subject factors. We used R’s stats package’s function t.test() for two-sample *t*-tests for comparisons of levels of between-subjects factors and paired *t*-tests for comparisons of levels of within-subjects factors. We considered results significant if *p* < 0.05 (two-sided). For effect sizes, we reported (partial) *η*^2^ as measures of explained variance.

### Mediation analyses

To conduct mediation analyses, we used the PROCESS R code (version 4.3)^58^, which is available online (http://processmacro.org/download.html). We modeled ordinary least squares regression path analyses and estimated direct and indirect effects in (serial) mediation analyses with one or two mediating variables. For instance, we investigated whether effects of interindividual differences in functional connectivity (independent variable) on interindividual differences in the extraction score (dependent variable) were mediated by differential engagement in perspective-taking and self-control processes (mediating variables, see Results sections for details). To test whether the indirect effects through the mediator(s) were statistically significant, we used 5′000 bootstrap samples to generate 95% bootstrap confidence intervals (95%-*CI*_*boot*_) for the indirect effect (with the value zero not contained in the interval indicating a significant indirect effect).

## Results

### Behavioral results

The histogram of participants’ extraction scores in Fig. 1A shows considerable interindividual differences in intergenerational sustainable behavior, with 90% of extraction scores falling within the interval [−1.11; 10.24]. Thus, most participants’ extraction scores ranged between about zero, indicating that a participant extracted an equal number of points irrespective of which generation was affected by exceeding the extraction threshold, and ten, indicating that a participant extracted 10 points more in trials affecting the next generation (*Gen*_*next*_) compared to trials affecting the present generation (*Gen*_*pres*_).

To better understand these extraction scores, we categorized participants into two behavioral types (Fig. 1B). Participants were categorized as sustainable if their median extraction in trials affecting the next generation was lower than or equal to 10 points and as unsustainable otherwise. This categorization resulted in 36 sustainable (mean extraction score ± *SD* = 0.46 ± 1.54) and 36 unsustainable participants (mean extraction score ± *SD* = 6.70 ± 3.28). Importantly, sustainable and unsustainable participants only differed in their behavior in *Gen*_*next*_ but not in *Gen*_*pres*_ trials, indicating that unsustainable (vs. sustainable) participants’ greater extraction scores were mainly driven by higher extraction behavior in trials affecting the next generation (see Fig. 1B). Thus, higher extraction scores represent more *un*sustainable behavior, while lower scores around zero represent more sustainable behavior towards the next generation.

We additionally tested whether altruistic, egoistic, hedonic, or biospheric values assessed by the SVS were associated with intergenerational sustainable behavior. Note that because one participant did not answer the online questionnaire, analyses regarding SVS values were based on a sample of 71 participants. A multiple regression of the extraction score on these values revealed that the more individuals endorsed altruistic values, the more sustainably they behaved towards the next generation (standardized regression coefficient *β* = −0.39, *t*(66) = −3.11, *p* = 0.003, *ΔR*^*2*^ = 0.12). None of the other three values was predictive of intergenerational sustainable behavior (all *p* > 0.05). Importantly, however, our functional activity and connectivity results (see below) hold when including altruistic values as covariate of no interest (see Supp. Analysis), indicating that these neural mechanisms uniquely explained variance in intergenerational sustainable behavior over and above participants’ altruistic values.

### Functional activity

**Figure 2:**
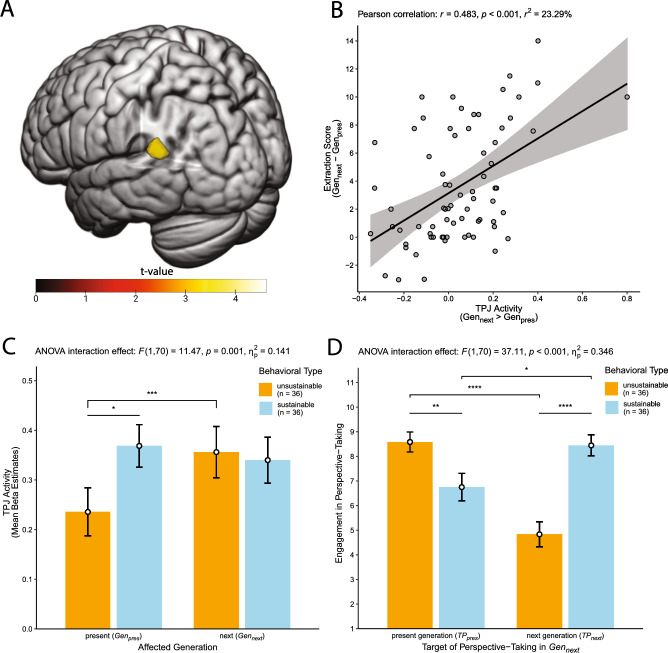
Functional activity in left TPJ predicts interindividual differences in intergenerational sustainability. Panel (**A**) shows a cluster in the left TPJ (projected on a rendered brain in MNI standard space) in which interindividual differences in functional activity (*Gen*_*next*_ > *Gen*_*pres*_) were associated with interindividual differences in the extraction score. Depicted is the statistical parametric map of the regression analysis with color-coded *t*-values, thresholded at whole-brain FDR-cluster corrected *p* < 0.05. The scatter plot in panel (**B**) shows that higher TPJ contrast estimates (*Gen*_*next*_ > *Gen*_*pres*_, extracted from the cluster shown in panel (**A**)) were associated with higher extraction scores (*Gen*_*next*_–*Gen*_*pres*_), indicating that greater TPJ activity in *Gen*_*next*_ relative to *Gen*_*pres*_ trials was associated with more *un*sustainable behavior. The scatter plot is complemented by a regression line of best fit with its 95% confidence interval and by the Pearson correlation coefficient *r*, *r*^2^ and the corresponding *p*-value. To provide a better understanding of this regression finding, panel (**C**) shows bar plots depicting extracted TPJ activity (mean beta estimates) separately for *Gen*_*next*_ and *Gen*_*pres*_ trials, broken down for the two behavioral sustainability types (unsustainable vs. sustainable). The plot shows that the association between TPJ activity and the extraction score is primarily driven by unsustainable (vs. sustainable) participants’ reduced TPJ activity in *Gen*_*pres*_ trials, whereas sustainable and unsustainable participants did not differ in TPJ activity in *Gen*_*next*_ trials. The bar plot in panel (**D**) illustrates that while the behavioral types did not differ in their overall engagement in perspective-taking in *Gen*_*next*_ trials, they differed considerably in whose perspective they took, that is in the target of perspective-taking in *Gen*_*next*_ trials. Sustainable (vs. unsustainable) participants more strongly took the perspective of others of the next generation, whereas unsustainable (vs. sustainable) participants more strongly took the perspective of others of the present generation. Error bars depict standard errors of the means and asterisks denote significant differences (* *p* < 0.05, ** *p* < 0.01, *** *p* < 0.005, **** *p* < 0.001) based on dependent and independent *t*-tests. For completeness, we also report the results of the significant two-way mixed ANOVA interaction effects. Brain images were generated using MRIcroGL (version 1.2.20211006, https://www.nitrc.org/projects/mricrogl).

We found (whole-brain FDR-cluster corrected at *p* < 0.05) that interindividual differences in functional activity and the extraction score were significantly associated in a hypothesized area of the brain, namely the left TPJ (x = −48, y = −46, z = 20, peak *t*-value = 4.78, see Fig. 2A), and in the right lingual gyrus (x = 12, y = −76, z = 4, peak *t*-value = 6.10). No additional findings emerged by applying a small-volume FWE- or FDR-cluster or -peak correction for bilateral MFG (which the DLPFC is part of), ACC, TPJ, and DMPFC. Contrary to expectations, however, greater left TPJ activity (*Gen*_*next*_ > *Gen*_*pres*_) was associated with higher extraction scores, that is the higher participants’ left TPJ activity was in trials affecting the next (relative to the present) generation, the more they behaved *un*sustainably towards the next generation (see Fig. 2B). We aimed at better understanding this activity pattern as a function of sustainable behavior by disentangling the difference values in activity and behavior. For this purpose, we extracted TPJ activity (mean beta estimates, thesholded at *p* < 0.005) separately for *Gen*_*next*_ and *Gen*_*pres*_ trials and plotted these extracted values broken down for the two behavioral sustainability types (see Fig. 2C). This revealed that the observed positive association was mainly driven by the fact that in trials affecting the present generation, unsustainable participants showed lower TPJ activity compared to sustainable participants. In contrast, sustainable and unsustainable participants did not show a differential TPJ activity pattern in *Gen*_*next*_ trials (*p* = 0.816).

Based on the TPJ’s known function in perspective-taking processes, these results might suggest that in *Gen*_*next*_ trials, sustainable and unsustainable participants equally engaged in perspective-taking. However, the behavioral sustainability types might still differ in *whose* perspective they took in trials affecting the next generation, that is they might differ in the target of perspective-taking in *Gen*_*next*_ trials. To investigate this possibility, we asked participants to indicate to what extent taking the perspective of others of the next (Target of Perspective-Taking: *TP*_*next*_) and of the present (*TP*_*pres*_) generation affected their decisions in *Gen*_*next*_ trials (see Methods for details). We conducted a two-way mixed ANOVA of this rated engagement in perspective-taking in *Gen*_*next*_ trials on the Target of Perspective-Taking in *Gen*_*next*_ (*TP*_*pres*_ vs. *TP*_*next*_) and Behavioral Type (unsustainable vs. sustainable). This analysis showed that the behavioral types did not differ in their overall engagement in taking the perspective of others in trials affecting the next generation (ANOVA main effect of Behavioral Type: *F*(1,70) = 3.03, *p* = 0.086, *η*_*p*_^*2*^ = 0.042), i.e. both types showed similarly strong perspective-taking in trials affecting the next generation. Importantly, however, the behavioral types considerably differed in whose perspective they took in trials affecting the next generation (ANOVA interaction effect: *F*(1,70) = 37.11, *p* < 0.001, *η*_*p*_^*2*^ = 0.346, see Fig. 2D). Sustainable (vs. unsustainable) participants showed a more next-generation oriented perspective-taking by more strongly taking the perspective of members of the next generation, whereas unsustainable (vs. sustainable) participants showed a more present-generation oriented perspective-taking by more strongly taking the perspective of members of the present generation.

In sum, we found that functional activity in TPJ was associated with interindividual differences in intergenerational sustainable behavior. However, in trials affecting the next generation, more sustainable and less sustainable participants did not differ in TPJ *activity* or overall engagement in perspective-taking. They differed considerably in whose perspective they took. Thus, we wondered whether the TPJ’s functional connectivity with other areas of the brain would reveal a differential pattern that helps to explain the interindividual differences in sustainable behavior and perspective-taking.

### Seed-based functional connectivity

**Figure 3:**
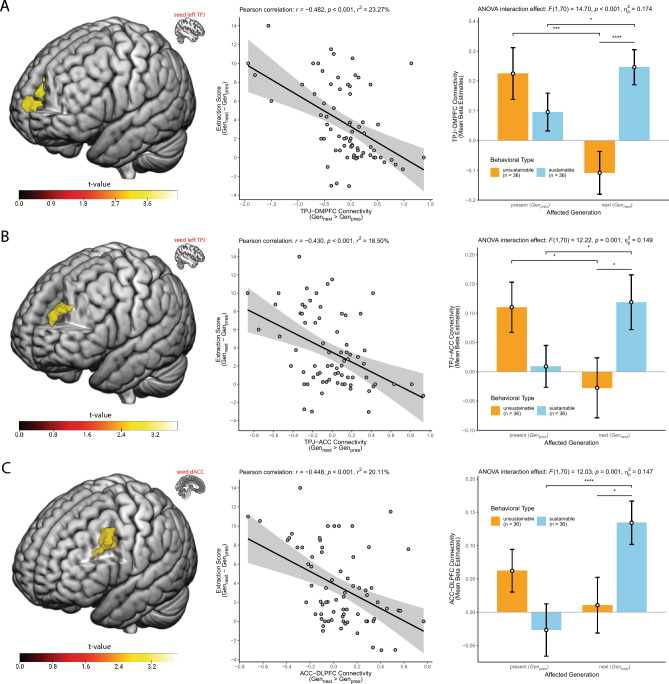
Seed-based functional connectivity predicts interindividual differences in intergenerational sustainability. Depicted are the functional connectivities seeded in the left TPJ (**A, B**) and seeded in the dorsal ACC (**C**) demonstrating a condition-specific (*Gen*_*next*_ > *Gen*_*pres*_) connectivity with the DMPFC (**A**), dorsal ACC (**B**) and left DLPFC (**C**) as a function of sustainable behavior, i.e., the stronger the increase was in functional connectivity in *Gen*_*next*_ compared to *Gen*_*pres*_, the more sustainably participants behaved, which is indicated by lower extraction score values (see Fig. 1 for details on the extraction score). Left: Statistical parametric maps of the regression analyses color-coded for the *t*-values as indicated by the color bar, thresholded at whole brain FDR-cluster corrected *p* < 0.05 and projected on a render brain in MNI space. Middle: Scatter plots showing the interindividual differences in extraction score (*Gen*_*next*_–*Gen*_*pres*_, y-axes) plotted against the interindividual differences in functional connectivity (*Gen*_*next*_ > *Gen*_*pres*_, x-axis) extracted from the depicted functional clusters (means of beta estimates). Regression lines of best fit with 95% confidence intervals and Pearson *r*, *r*^2^ and the corresponding *p*-values are displayed. Right: For an improved understanding of the regression findings, bar plots show the disentangled connectivity values separately for *Gen*_*next*_ and *Gen*_*pres*_ and broken down for the two behavioral types (derived from the extraction behavior, see Fig. 1). Error bars depict standard errors of the means and asterisks denote significant differences (* *p* < 0.05, ** *p* < 0.01, *** *p* < 0.005, **** *p* < 0.001) based on dependent and independent *t*-tests. For completeness, we also report the results of the significant two-way mixed ANOVA interaction effect. Brain images were generated using MRIcroGL (version 1.2.20211006, https://www.nitrc.org/projects/mricrogl).

Connectivity analyses seeded in the left TPJ (identified in the activity pattern analyses reported above) revealed a distinctive connectivity pattern of the left TPJ with four other areas of the brain. More precisely, we found (whole-brain FDR-cluster corrected at *p* < 0.05) that the left TPJ showed increased functional connectivity (*Gen*_*next*_ > *Gen*_*pres*_) with the DMPFC (x = 6, y = 65, z = 11, peak *t*-value: 4.51, see Fig. 3A), the dorsal ACC (x = -3, y = 35, z = 29, peak *t*-value = 3.79, see Fig. 3B), the left anterior insula/fronto-insular cortex (x = −42, y = 23, z = −13, peak *t*-value = 4.72, see Supp. Fig. S1A) and the hippocampus/parahippocampus (x = 27, y = −28, z = −19, peak *t*-value = 4.25, see Supp. Fig. S1B) as a function of sustainable behavior (measured with the extraction score). That is, the stronger the increase was in functional connectivity (*Gen*_*next*_ > Gen_pres_) between these areas, the more sustainably a participant behaved towards the next generation as indexed by extraction score values near zero (see scatterplots in Fig. 3A/B and Supp. Fig. S1a/b). To better understand the observed connectivity pattern as a function of sustainable behavior (i.e., to disentangle the difference values in connectivity and behavior), we extracted the connectivity values (thresholded at *p* < 0.001 for TPJ-DMPFC connectivity and at *p* < 0.005 for TPJ-ACC connectivity) in the significant clusters separately for the two conditions and plotted these values, broken down for the two behavioral types. As can be seen in the bar plots in Fig. 3A/B and Supp. Fig. S1a/b), the observed regression effects are mainly driven by the distinct connectivity pattern in trials affecting the next generation, where sustainable (vs. unsustainable) participants showed an enhanced functional connectivity between left TPJ and all four reported regions. However, we would also like to point out that in some regions (e.g., in the dorsal ACC) one can also observe a (partly) reversed pattern in trials affecting the present generation, where unsustainable (vs. sustainable) participants demonstrated an enhanced functional connectivity on a descriptive (albeit not statistically significant) level. We address this issue in the Supp. Discussion.

No other areas in the brain showed a functional connectivity pattern with the left TPJ as a function of sustainable behavior, also not if we apply small-volume corrections for our hypothesized regions of interests. In this regard, it is interesting to note that there seems to be no such functional connectivity pattern between the left TPJ and dorsolateral areas of the prefrontal cortex, as hypothesized. However, as outlined in the introduction, the dorsal ACC and dorsolateral areas of the prefrontal cortex often act together to solve a decision conflict, i.e., the dorsal ACC monitors and signals conflicts and recruits the DLPFC to solve them^32–34^. Thus, we wondered whether the dorsal ACC (identified in the connectivity analysis seeded in the left TPJ reported above) might demonstrate a condition-specific connectivity pattern with the DLPFC as a function of sustainable behavior. Connectivity analyses seeded in the dorsal ACC revealed (whole-brain FDR-cluster corrected at *p* < 0.05) an increased functional connectivity (*Gen*_*next*_ > *Gen*_*pres*_) of the dorsal ACC with an area of the left (caudal) DLPFC (x = −36, y = 8, z = 41, peak *t*-value: 3.83, see Fig. 3C) as a function of sustainable behavior. That is, the stronger the increase was in functional connectivity (*Gen*_*next*_ > Gen_pres_) between the dorsal ACC and left DLPFC, the more sustainably participants behaved towards the next generation as indexed by extraction score values near zero (see scatterplots in Fig. 3C). To disentangle the difference values (in connectivity and behavior), we again extracted the connectivity values (thresholded at *p* < 0.005) in the significant clusters separately for the two conditions and plotted these values, broken down for the two behavioral types. As can be seen in the bar plot in Fig. 3C, the observed regression effect is mainly driven by the distinct connectivity pattern in trials affecting the next generation, where sustainable (vs. unsustainable) participants showed an enhanced functional connectivity between dorsal ACC and left DLPFC. No other area showed a significant condition-specific functional connectivity with the dorsal ACC as a function of sustainable behavior, neither whole-brain nor small-volume corrected.

### Mediators between functional connectivity networks and intergenerational sustainability

Our findings revealed that the functional connectivity between key areas involved in perspective-taking (TPJ-DMPFC connectivity) and self-control (ACC-DLPFC connectivity) seem to play an important role in intergenerational sustainability. Thus, in a final set of mediation analyses we tested whether the functional connectivity within these networks indeed affected intergenerational sustainability by influencing social-cognitive and self-control related processes.

**Figure 4:**
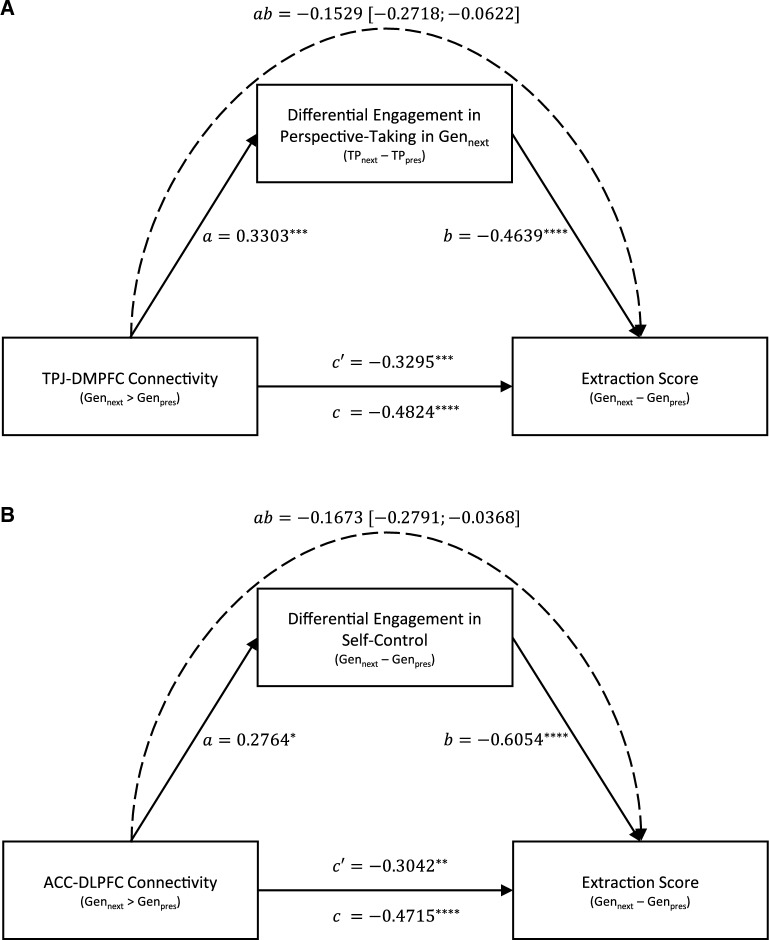
Perspective-taking and self-control related processes as mediators between functional connectivity networks and intergenerational sustainability. Depicted in (**A**) is the path diagram of the mediation analysis demonstrating that differential engagement in perspective-taking mediates the impact of TPJ-DMPFC connectivity on the extraction score, i.e., stronger functional connectivity between TPJ and DMPFC (*Gen*_*next*_ > *Gen*_*pres*_) predicted more next-generation oriented perspective-taking (*TP*_*next*_–*TP*_*pres*_), which in turn was associated with increased intergenerational sustainable behavior (lower extraction scores). Depicted in (**B**) is the path diagram of the mediation analysis demonstrating that differential engagement in self-control (i.e., differential efforts in resting temptations) mediate the impact of ACC-DLPFC connectivity on the extraction score, i.e., stronger functional connectivity between ACC and DLPFC (*Gen*_*next*_ > *Gen*_*pres*_) predicted higher efforts to resist temptation (*Gen*_*next*_–*Gen*_*pres*_), which in turn was associated with increased intergenerational sustainable behavior. Note that in the depicted mediation analyses, all requirements for mediation effects are satisfied: All (required) paths and the indirect effects are significant. The path diagrams depict standardized linear regression coefficients and asterisks denote significant effects: * *p* < 0.05, ** *p* < 0.01, *** *p* < 0.005, **** *p* < 0.001. The intervals of the indirect effects (*ab*) represent 95% confidence intervals calculated using 5′000 bootstrap samples (with the value zero not contained in the intervals indicating significant indirect effects). *c* = total effect, *c’* = direct effect.

Participants indicated to what extent taking the perspective of others of the next (Target of Perspective-Taking: *TP*_*next*_) and of the present (*TP*_*pres*_) generation affected their decisions in trials affecting the next generation (see Methods section for details). As illustrated in Fig. 2D and Supp. Fig. S2a, sustainable (vs. unsustainable) participants demonstrated a more next-generation oriented perspective-taking. Based on these ratings, we calculated participants’ differential engagement in perspective-taking in *Gen*_*next*_ trials (*TP*_*next*_–*TP*_*pres*_, see Methods section). Hence, higher positive values indicated that participants more strongly engaged in taking the perspective of others of the next generation, whereas lower negative values indicated that participants more strongly engaged in taking the perspective of others of the present generation, and values around zero represented that participants engaged in taking the perspective of others of both generations in a balanced way. We ran a mediation analysis to test whether the observed differential engagement in perspective-taking mediated the impact of TPJ-DMPFC connectivity on intergenerational sustainable behavior (measured with the extraction score). We found (see Fig. 4A) that stronger TPJ-DMPFC connectivity (*Gen*_*next*_ > *Gen*_*pres*_) predicted more next-generation oriented perspective-taking, which in turn was associated with increased intergenerational sustainable behavior (lower extraction scores). Importantly, all critical paths (see Fig. 4A) and the indirect effect were significant (product of standardized regression coefficients: *ab* = −0.153, 95%-*CI*_*boot*_ = [−0.272; -0.062]).

Participants additionally indicated to what extent they were tempted to extract more than 10 points and to what extent they tried to resist this temptation in *Gen*_*next*_ and *Gen*_*pres*_ trials (see Methods section for details). Based on these ratings, we again calculated difference scores (*Gen*_*next*_–*Gen*_*pres*_). Hence, higher positive values in differential effort to resist temptation indicated that participants more strongly tried to resist the temptation in *Gen*_*next*_ trials, whereas lower negative values indicated that participants more strongly tried to resist the temptation in *Gen*_*pres*_ trials, and values near zero represented equal efforts to resist temptations irrespective of which generation was affected. In fact, sustainable participants reported an equal (and high) effort to resist temptation in both conditions (*Gen*_*next*_ and *Gen*_*pres*_), whereas unsustainable participants reported a significantly lower efforts to resist temptation in *Gen*_*next*_ (vs. *Gen*_*pres*_) trials (see Supp. Fig. S2b for details). Hence, we ran a mediation analysis to test whether differential effort to resist temptation mediated the impact of ACC-DLPFC connectivity on intergenerational sustainability. Crucially, we controlled in this analysis for differential temptation because (failed) self-control processes depend on the experience of temptation in the first place, which is why researchers should control for the degree to which participants are actually tempted by certain stimuli^51,52^. We found (see Fig. 4B) that stronger ACC-DLPFC connectivity (*Gen*_*next*_ > *Gen*_*pres*_) predicted higher efforts to resist temptation in trials affecting the next (vs. present) generation, which in turn was associated with increased intergenerational sustainable behavior (lower extraction scores). Importantly, all critical paths (see Fig. 4B) and the indirect effect were significant (*ab* = −0.167, 95%-*CI*_*boot*_ = [−0.279; −0.037]). Please note that mediation results hold if efforts to resist temptation only in trials affecting the next generation is used as mediator (and not the difference value) and if the mediation is run without differential temptation as covariate (note that differential temptation and extraction score were not correlated, see Supp. Fig. S2c for further details). Finally, please see Supp. Fig. S3 for a serial mediation analysis investigating a tentative and speculative integrative model of both processes (perspective-taking and self-control).

## Discussion

What mental processes and neural mechanisms drive interindividual differences in intergenerational sustainable behavior? Here, we assessed neural responses during decision-making in a consequential intergenerational sustainability dilemma game, in which successive groups of participants (i.e., generations) could extract points from a common pool under two experimental conditions (*Gen*_*next*_ vs. *Gen*_*pres*_). Modeling the critical contingencies of intergenerational sustainability, overharvesting of the pool considerably reduced either the payoff of the next (*Gen*_*next*_) or the present generation (*Gen*_*pres*_, control condition). The difference in extracted points in *Gen*_*next*_ minus *Gen*_*pres*_ (labelled extraction score) served as a measure of interindividual differences in intergenerational sustainable behavior. Results showed that differences in the extraction score were associated with differences in activity and connectivity within the mentalizing network (TPJ activity, TPJ-DMPFC connectivity), connectivity between the mentalizing and cognitive control network (TPJ-ACC connectivity), and connectivity within the cognitive control network (ACC-DLPFC connectivity).

The TPJ, a core node of the mentalizing network, is involved in social cognition, including perspective-taking, theory of mind, and self-other distinction^25,29,59–62^. For instance, TPJ activity has been related to overcoming egoism bias during decisions affecting socially distant others in a single-generation social discounting task^63^. Moreover, causally enhancing the cortical excitability of the TPJ has been shown to increase sustainable behavior in an intergenerational sustainability dilemma game, supposedly by enhancing intergenerational mentalizing^48^. Thus, we expected that greater TPJ activity would be associated with more sustainable behavior due to increased perspective-taking with future others. While results confirmed that interindividual differences in TPJ activity were indeed related to differences in intergenerational sustainability, the association’s direction was unexpected at first glance: Enhanced TPJ activity in *Gen*_*next*_ (vs. *Gen*_*pres*_) trials was associated with *less* sustainable behavior. Importantly, however, a closer inspection revealed that this association was driven by unsustainable (vs. sustainable) participants’ reduced TPJ activity in *Gen*_*pres*_ trials (control condition), whereas no difference in TPJ activity occurred in *Gen*_*next*_ trials. This aligns with the finding that unsustainable and sustainable participants did not differ in *overall* engagement in reported perspective-taking in *Gen*_*next*_ trials. However, they differed considerably in *whose* perspective they took (i.e., *targets* of perspective-taking). Unsustainable participants’ perspective-taking was more present-generation oriented, whereas sustainable participants’ perspective-taking was more balanced or next-generation oriented. Yet, these behaviorally relevant interindividual variability in differential engagement in perspective-taking (present- vs. next-generation oriented) seemed not to be encoded in the local activity pattern of the TPJ. Taking the perspective of others is a difficult task, which presumably becomes even more challenging if the targets are unknown, future strangers. Thus, such intergenerational perspective-taking might require a more elaborate communication between multiple nodes of the mentalizing network.

In support of this idea, we found that greater TPJ-DMPFC connectivity in *Gen*_*next*_ (vs. *Gen*_*pres*_) trials was associated with more sustainable behavior and that this effect was mediated by more next-generation oriented perspective-taking. As core nodes of the mentalizing network, the TPJ and DMPFC are known to be tightly interconnected, both structurally and functionally^62,64–70^. Differences in structural and functional TPJ-DMPFC connectivity have been found to drive interindividual differences in perspective-taking related social behaviors in single-generation contexts, including intergroup situations, trust games, and third-party punishment paradigms^30,71,72^. The present findings extend the importance of TPJ-DMPFC connectivity for perspective-taking in single-generation contexts to intergenerational behavior. Based on the known functions of the mentalizing network and on our mediation analysis, we therefore interpret increased TPJ-DMPFC functional connectivity as indexing more elaborate communication within the mentalizing network, enabling individuals to better engage in taking the perspective of future others. This increased intergenerational mentalizing, i.e., thinking about the needs and mental states of others in the future, might activate the goal not to curtail the outcomes of future others, which might ultimately motivate sustainable behavior. Once activated, this goal likely conflicts with the desire to maximize benefits for oneself in the present, a characteristic feature of intergenerational sustainability dilemmas^13,21^. Such desire-goal conflicts may manifest in temptations^51,52,73,74^, which need to be monitored and overcome to behave sustainably. Thus, the question arises: Which neural mechanisms could potentially account for why certain individuals manage to effectively navigate these conflicts, resisting temptations and engaging in sustainable behavior, while others do not?

We found that more sustainable behavior was associated with increased TPJ‑ACC and ACC‑DLPFC connectivity in *Gen*_*next*_ (vs. *Gen*_*pres*_) trials. The ACC and DLPFC are thought to constitute a cognitive control network, in which the ACC monitors conflicts based on inputs it receives from diverse brain areas and sends control signals to the DLPFC, where self-control processes are implemented accordingly^32,34,75–78^. Functioning of the ACC and DLPFC is known to be crucially involved in a wide range of single-generation contexts requiring self-control, including intertemporal choice, intergroup situations, and social dilemmas^42,79–84^. The TPJ is one of the brain regions providing the ACC with inputs for conflict monitoring through known structural and functional interconnections^34,65,85,86^. Thus, greater TPJ-ACC connectivity in *Gen*_*next*_ (vs. *Gen*_*pres*_) trials might suggest heightened communication *between* the mentalizing and cognitive control network, possibly indexing a more elaborate monitoring and processing of potential conflicts arising from the trade-off between benefitting oneself or future others. Moreover, increased ACC-DLPFC connectivity in *Gen*_*next*_ (vs. *Gen*_*pres*_) trials might represent greater communication *within* the cognitive control network, enabling a more intricate engagement in self-control processes (allocation and execution of cognitive control) needed to overcome selfish, unsustainable temptations. This interpretation is further substantiated by our finding that the effect of greater ACC-DLPFC connectivity in *Gen*_*next*_ (vs. *Gen*_*pres*_) trials on more sustainable behavior was mediated by increased reported engagement in self-control (i.e., efforts to resist temptations) in trials affecting the next (vs. present) generation.

Cumulatively, these findings integrate into a picture in which differences in the neural communication within and between the mentalizing and cognitive control network drive interindividual differences in intergenerational sustainable behavior. Based on these networks’ known functions and supported by our single mediator and multiple serial mediator analyses, our results speak for a central role of perspective-taking and self-control processes during intergenerational sustainable decision-making. This resonates with and synthesizes theories and behavioral, non-neuroscientific studies suggesting that perspective-taking^21,87–89^ and cognitive control^90–95^ play important roles in sustainable behavior. Here, we complement and extend this research by assessing neural responses during intergenerational decision-making and providing first evidence that task-dependent neural mechanisms related to perspective-taking and self-control explain interindividual differences in intergenerational sustainability.

We would like to emphasize that differences in other processes than the ones discussed thus far might also contribute to behavioral heterogeneity in intergenerational sustainability. In fact, we found, beyond the scope of our hypotheses, that greater functional connectivity between the TPJ and anterior insula (aINS) and between the TPJ and the hippocampus (HC) was associated with more sustainable behavior (Supp. Fig. S1 and Supp. Discussion). The aINS is known to be involved in affective processes, including empathy^82,96^, and the HC is known to play a crucial role in prospective thinking^97,98^. Thus, these results suggest that differences in processes involving empathy and prospective thinking might be additional drivers of interindividual differences in intergenerational sustainable behavior (for a more detailed discussion, please check Supp. Discussion), which also aligns with research showing the importance of these processes in single-generation intergroup situations^99–102^ and sustainable behavior^103^. Future research focusing on these and other additional potential processes could deepen our understanding of the neural mechanisms driving heterogeneity in intergenerational sustainable behavior.

Our results fit in and substantially extend the few existing neuroscientific studies on intergenerational sustainability. In a recent neural trait study using the same behavioral paradigm as the one deployed here, we found that sustainable participants were marked by greater cortical thickness of the DMPFC and left DLPFC compared to unsustainable participants, and that these effects were mediated by increased next-generation oriented perspective-taking and self-control, respectively^11^. Notably, these findings were in close proximity to the TPJ-DMPFC and ACC-DLPFC functional connectivity findings in the present study. Thus, we found a considerable congruence in brain regions despite distinctively different neural measures assessed in the two studies (task-independent, “offline” cortical thickness vs. task-dependent, “online” functional activity and connectivity during decision-making). Together, these results suggest that trait-like structural and state-like functional differences in the same brain regions related to perspective-taking and self-control drive interindividual differences in intergenerational sustainable behavior. This might be interpreted as evidence that both, the general capacity for as well as actual engagement in perspective-taking and self-control processes during decision-making explain interindividual differences in intergenerational sustainability.

The present study might also shed new light on results from two recent brain stimulation studies on intergenerational sustainable behavior. First, Langenbach and colleagues^49^ found that inhibiting the right DLPFC by means of continuous theta-burst transcranial magnetic stimulation (TMS) did not alter behavior in an intergenerational sustainability dilemma game very similar to the one used here. As a potential explanation for this missing effect, the authors discussed that, maybe, not the right, but the left DLPFC is causally involved in self-control related intergenerational sustainable behavior, given evidence that inhibition of the left, but not the right, DLPFC reduces self-control in the context of single-generation intertemporal choice^42^. In support of this line of thought, we found that functional connectivity between the ACC and the left DLPFC was associated with individual differences in intergenerational sustainable behavior. Second, Langenbach and colleagues^48^ showed that applying high-definition transcranial direct current stimulation over the TPJ causally enhanced sustainable behavior in the same intergenerational task as in the previous TMS study. At first, this might suggest that local TPJ activity causally induces intergenerational mentalizing giving rise to intergenerational sustainable behavior. However, we find that local activity of a single node of the mentalizing network falls short of explaining behaviorally relevant differences in future-generation oriented perspective-taking. Importantly, brain stimulation techniques are known to alter brain function not only locally but also in distributed networks^104–107^, and transcranial magnetic and direct current stimulation of the TPJ has been found to alter TPJ-DMPFC and TPJ-ACC functional connectivity^108,109^. Thus, one might speculate that the TPJ stimulation effect reported by Langenbach and colleagues^48^ might not (solely) be due to the enhancement of local TPJ activity but to increased functional connectivity of the TPJ with other nodes of the mentalizing and cognitive control network. Future studies combining brain stimulation and imaging techniques in sustainability research could enhance our understanding of the neural networks causally involved in intergenerational sustainable behavior.

We recognize certain limitations of our study that we recommend considering and addressing in future research. In designing our behavioral paradigm, we aimed at capturing the complexity of real intergenerational sustainable behavior. However, mimicking intergenerational behavior between real generations in a controlled setting is extremely challenging, if not practically impossible. To overcome this limitation, researchers have successfully designed paradigms approximating the key features and contingencies of intergenerational behavior. This approach was first introduced in seminal work by Hauser and colleagues^44^ and has since been applied in various behavioral studies^21,45–47^. Similar to these studies, our paradigm consisted of successive groups that were separated by a temporal gap and extracted resources from a common pool, whose overharvesting affected the payoff of the next or present group. Crucially, this paradigm modelled important aspects of intergenerational sustainability dilemmas. These aspects included the social and temporal distance between benefactors and beneficiaries, the temporal delay between actions and consequences, the unidirectional nature of the interactions, and the absence of reciprocity. As argued previously^21,44–49^, incorporating these features allows to consider the “next group” as a valid proxy for a “future generation”, enabling the paradigm to reasonably capture intergenerational contingencies within the confines of a well-controlled laboratory environment. Nevertheless, such paradigms optimized for laboratory settings necessarily remain approximations of real-world intergenerational behaviors. Future studies developing practical solutions for investigating behaviors between real generations could advance sustainability research.

The present study deliberately focused on the interplay of social and temporal dimensions distinctively characterizing intergenerational sustainable behavior. When viewed in isolation, these dimensions also constitute components of some single-generation dilemmas (e.g., intergroup situations and intertemporal choice). It is a different question whether and how behavior and neural processes in these single-generation dilemmas relate to intergenerational sustainability. Future research could investigate such potential relationships by using different designs optimized for these questions. For instance, future studies could employ full factorial designs crossing different levels of social and temporal dimensions, shedding light on the specificity of neural underpinnings of intergenerational sustainability dilemmas compared to single-generation dilemmas.

We acknowledge that our functional neuroimaging findings do not definitively distinguish between a capacity-based interpretation and a motivational-based interpretation. This limitation is inherent to many neuroimaging studies and highlights the complexity of interpreting neural activation and connectivity patterns. Our findings demonstrate which specific brain regions are more active or functionally connected when participants make intergenerationally sustainable decisions compared to unsustainable decisions. However, these findings alone do not definitively answer the question of whether unsustainable participants are incapable or unwilling (or both) to behave in an intergenerationally sustainable manner.

Our connectivity findings consistently reflect a similar pattern across the reported regions, prompting consideration of the potential influence of a broader network connectivity effect. While the selectivity of our connectivity findings (e.g., the ACC exclusively exhibits this pattern with the left DLPFC) mitigate the plausibility of such an effect, we recognize that a complete exclusion is difficult. Addressing this uncertainty will require future neuroimaging studies dedicated to exploring intergenerational sustainability.

In conclusion, the present study identified differences in task-dependent functional activity and connectivity within and between brain networks engaged in perspective-taking and self-control processes as drivers of interindividual differences in intergenerational sustainable behavior. By combining a consequential behavioral task with the assessment of objective neural responses during decision-making, the study answers intensifying calls for measuring actual (vs. hypothetical) behavior and leveraging neuroscience for sustainability research^6–10,110^. Importantly, we view our work as contributing to basic research, and we therefore do not claim that our study has direct policy relevance. The challenge of intergenerational sustainability cannot be solved by neuroscience alone. However, neuroscience can uniquely contribute to the transdisciplinary efforts to advance sustainability research by providing exclusive access to mental processes and neural mechanisms giving rise to intergenerational sustainable behavior and its interindividual differences.

### Supplementary Information

Supplementary Information.

## Data Availability

The data that support the findings of this study are available from the corresponding author upon reasonable request.
