# Publisher Correction: Neural mechanisms underlying interindividual differences in intergenerational sustainable behaviour

**DOI:** 10.1038/s41598-023-47161-1

**Published:** 2023-11-27

**Authors:** Thomas Baumgartner, Emmanuel Guizar Rosales, Daria Knoch

**Affiliations:** 1 Department of Social Neuroscience and Social Psychology, Institute of Psychology, University of Bern, Fabrikstrasse 8, CH-3012 Bern, Switzerland; 2 Translational Imaging Center (TIC), Swiss Institute for Translational and Entrepreneurial Medicine, Bern, Switzerland

Correction to: *Scientific Reports* 10.1038/s41598-023-44250-z, published online 13 October 2023

The original version of this Article contained errors. In Figures 2 and 3 the confidence intervals were inadvertently omitted during typesetting from all scatterplots.

The original Figures 2 and 3 and accompanying legend appear below.

**Figure 2:**
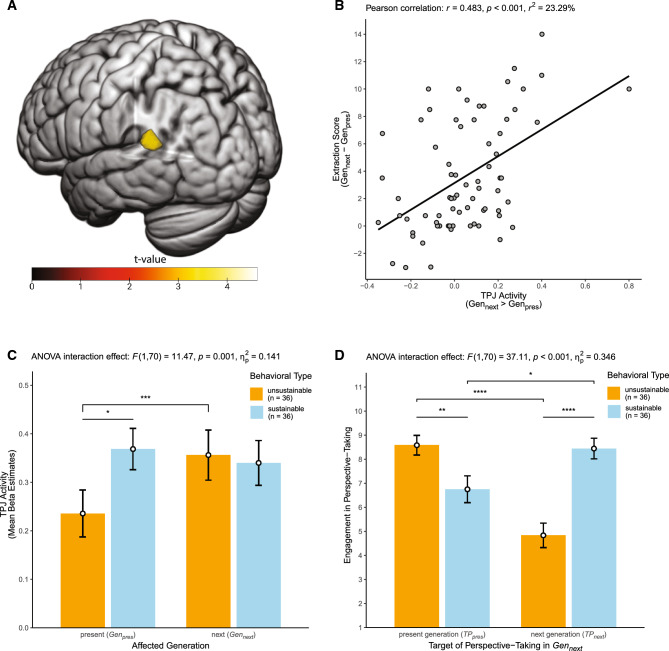
Functional activity in left TPJ predicts interindividual differences in intergenerational sustainability. Panel (**A**) shows a cluster in the left TPJ (projected on a rendered brain in MNI standard space) in which interindividual differences in functional activity (*Gen*_*next*_ > *Gen*_*pres*_) were associated with interindividual differences in the extraction score. Depicted is the statistical parametric map of the regression analysis with color-coded *t*-values, thresholded at whole-brain FDR-cluster corrected *p* < 0.05. The scatter plot in panel (**B**) shows that higher TPJ contrast estimates (*Gen*_*next*_ > *Gen*_*pres*_, extracted from the cluster shown in panel (**A**)) were associated with higher extraction scores (*Gen*_*next*_–*Gen*_*pres*_), indicating that greater TPJ activity in *Gen*_*next*_ relative to *Gen*_*pres*_ trials was associated with more *un*sustainable behavior. The scatter plot is complemented by a regression line of best fit with its 95% confidence interval and by the Pearson correlation coefficient *r*, *r*^2^ and the corresponding *p*-value. To provide a better understanding of this regression finding, panel (**C**) shows bar plots depicting extracted TPJ activity (mean beta estimates) separately for *Gen*_*next*_ and *Gen*_*pres*_ trials, broken down for the two behavioral sustainability types (unsustainable vs. sustainable). The plot shows that the association between TPJ activity and the extraction score is primarily driven by unsustainable (vs. sustainable) participants’ reduced TPJ activity in *Gen*_*pres*_ trials, whereas sustainable and unsustainable participants did not differ in TPJ activity in *Gen*_*next*_ trials. The bar plot in panel (**D**) illustrates that while the behavioral types did not differ in their overall engagement in perspective-taking in *Gen*_*next*_ trials, they differed considerably in whose perspective they took, that is in the target of perspective-taking in *Gen*_*next*_ trials. Sustainable (vs. unsustainable) participants more strongly took the perspective of others of the next generation, whereas unsustainable (vs. sustainable) participants more strongly took the perspective of others of the present generation. Error bars depict standard errors of the means and asterisks denote significant differences (*p < 0.05, **p < 0.01, ***p < 0.005, ****p < 0.001) based on dependent and independent *t*-tests. For completeness, we also report the results of the significant two-way mixed ANOVA interaction effects. Brain images were generated using MRIcroGL (version 1.2.20211006, https://www.nitrc.org/projects/mricrogl).

**Figure 3:**
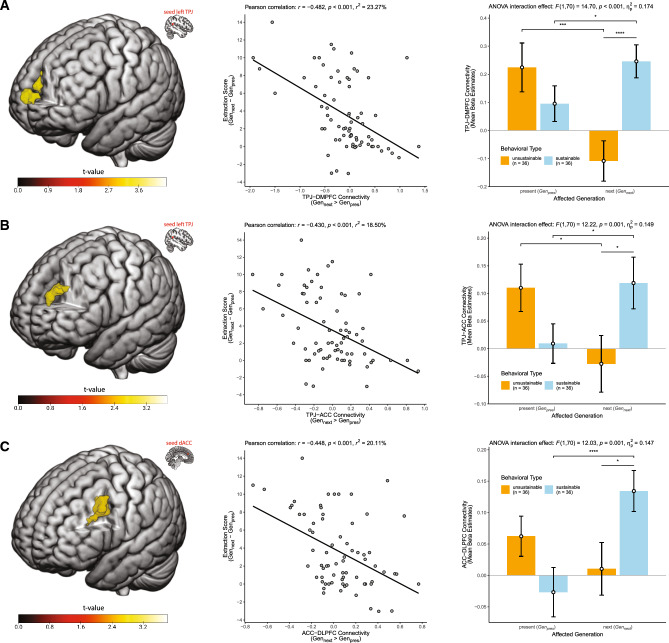
Seed-based functional connectivity predicts interindividual differences in intergenerational sustainability. Depicted are the functional connectivities seeded in the left TPJ (**A**,**B**) and seeded in the dorsal ACC (**C**) demonstrating a condition-specific (*Gen*_*next*_ > *Gen*_*pres*_) connectivity with the DMPFC (**A**), dorsal ACC (**B**) and left DLPFC (**C**) as a function of sustainable behavior, i.e., the stronger the increase was in functional connectivity in *Gen*_*next*_ compared to *Gen*_*pres*_, the more sustainably participants behaved, which is indicated by lower extraction score values (see Fig. 1 for details on the extraction score). Left: Statistical parametric maps of the regression analyses color-coded for the *t*-values as indicated by the color bar, thresholded at whole brain FDR-cluster corrected *p* < 0.05 and projected on a render brain in MNI space. Middle: Scatter plots showing the interindividual differences in extraction score (*Gen*_*next*_–*Gen*_*pres*_, y-axes) plotted against the interindividual differences in functional connectivity (*Gen*_*next*_ > *Gen*_*pres*_, x-axis) extracted from the depicted functional clusters (means of beta estimates). Regression lines of best fit with 95% confidence intervals and Pearson *r*, *r*^2^ and the corresponding *p*-values are displayed. Right: For an improved understanding of the regression findings, bar plots show the disentangled connectivity values separately for *Gen*_*next*_ and *Gen*_*pres*_ and broken down for the two behavioral types (derived from the extraction behavior, see Fig. 1). Error bars depict standard errors of the means and asterisks denote significant differences (*p < 0.05, **p < 0.01, ***p < 0.005, ****p < 0.001) based on dependent and independent *t*-tests. For completeness, we also report the results of the significant two-way mixed ANOVA interaction effect. Brain images were generated using MRIcroGL (version 1.2.20211006, https://www.nitrc.org/projects/mricrogl).

The original Article has been corrected.

