# Supplementary material for: Neural mechanisms underlying interindividual differences in intergenerational sustainable behavior
Source: Sci Rep. 2023 Oct 13;13:17357. doi: 10.1038/s41598-023-44250-z (PMC10575884; doi:10.1038/s41598-023-44250-z)
Supplement: Supplementary file 1 — Supplementary Information. [file 41598_2023_44250_MOESM1_ESM.docx]

**Supplementary information for**

Neural mechanisms underlying interindividual differences in intergenerational sustainable behavior

Thomas Baumgartner^a,b,1^, Emmanuel Guizar Rosales^a,b,1^, and Daria Knoch^a,b^

^a^ Department of Social Neuroscience and Social Psychology, Institute of Psychology, University of Bern, Switzerland

^b^ Translational Imaging Center (TIC), Swiss Institute for Translational and Entrepreneurial Medicine, Bern, Switzerland

^1^ The first two authors contributed equally to this work.

**Corresponding author:**

Thomas Baumgartner (thomas.baumgarter@unibe.ch)

**Inventory of supplementary material:**

**Supplementary Figure S1 & S2 & S3**

**Supplementary Analysis**

**Supplementary Discussion**

**References**

**
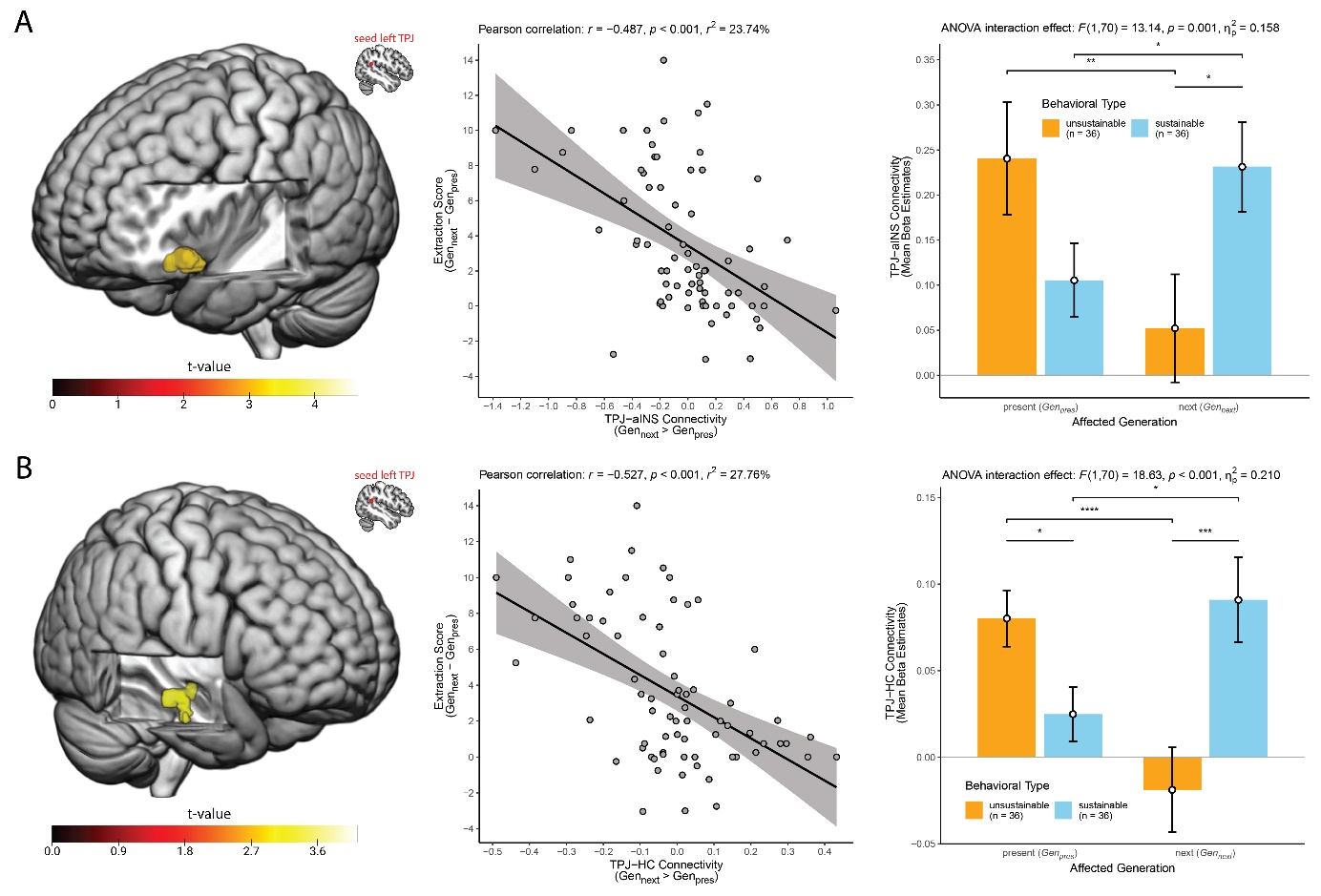
**

***Suppl. Fig. S1.* Seed-based functional connectivity predicts interindividual differences in intergenerational sustainability.** Depicted are the functional connectivities seeded in the left TPJ demonstrating a condition-specific (*Gen_next_* > *Gen_pres_*) connectivity with the **(A)** anterior insula/fronto-insular cortex (aINS) and **(B)** hippocampus/parahippocampus (HC) as a function of sustainable behavior, i.e., the stronger the increase was in functional connectivity in *Gen_next_* compared to *Gen_pres_*, the more participants behaved sustainably, which is indicated by low extraction score values (see Fig. 1 for details on the extraction score). **Left**: Statistical parametric maps of the regression analyses color-coded for the *t*-values as indicated by the color bar, thresholded at whole brain FDR-cluster corrected *p* < 0.05 and projected on a render brain in MNI space. **Middle:** Scatter plots showing the interindividual differences in extraction score (*Gen_next_* > *Gen_pres_*, y-axes) plotted against the interindividual differences in functional connectivity (*Gen_next_* > *Gen_pres_*, x-axis) extracted from the depicted functional clusters (means of beta estimates, thresholded at *p* < 0.001). Regression lines represent lines of best fit with 95% confidence intervals and Pearson *r,* *r*^2^, and the corresponding *p*-values are reported. **Right:** For an improved understanding of the regression findings, bar plots show the disentangled connectivity values separately for *Gen_next_* and *Gen_pres_* and broken down for the two behavioral types (derived from the extraction behavior, see Fig. 1). Error bars depict standard errors of the means and asterisks denote significant differences (* *p* < 0.05, ** *p* < 0.01, *** *p* < 0.005, **** *p* < 0.001) based on dependent and independent *t*-tests. For completeness, we also reported the results of the significant two-way mixed ANOVA interaction effects between Affected Generation and Behavioral Types. Brain images were generated using MRIcroGL (version 1.2.20211006, https://www.nitrc.org/projects/mricrogl).

**
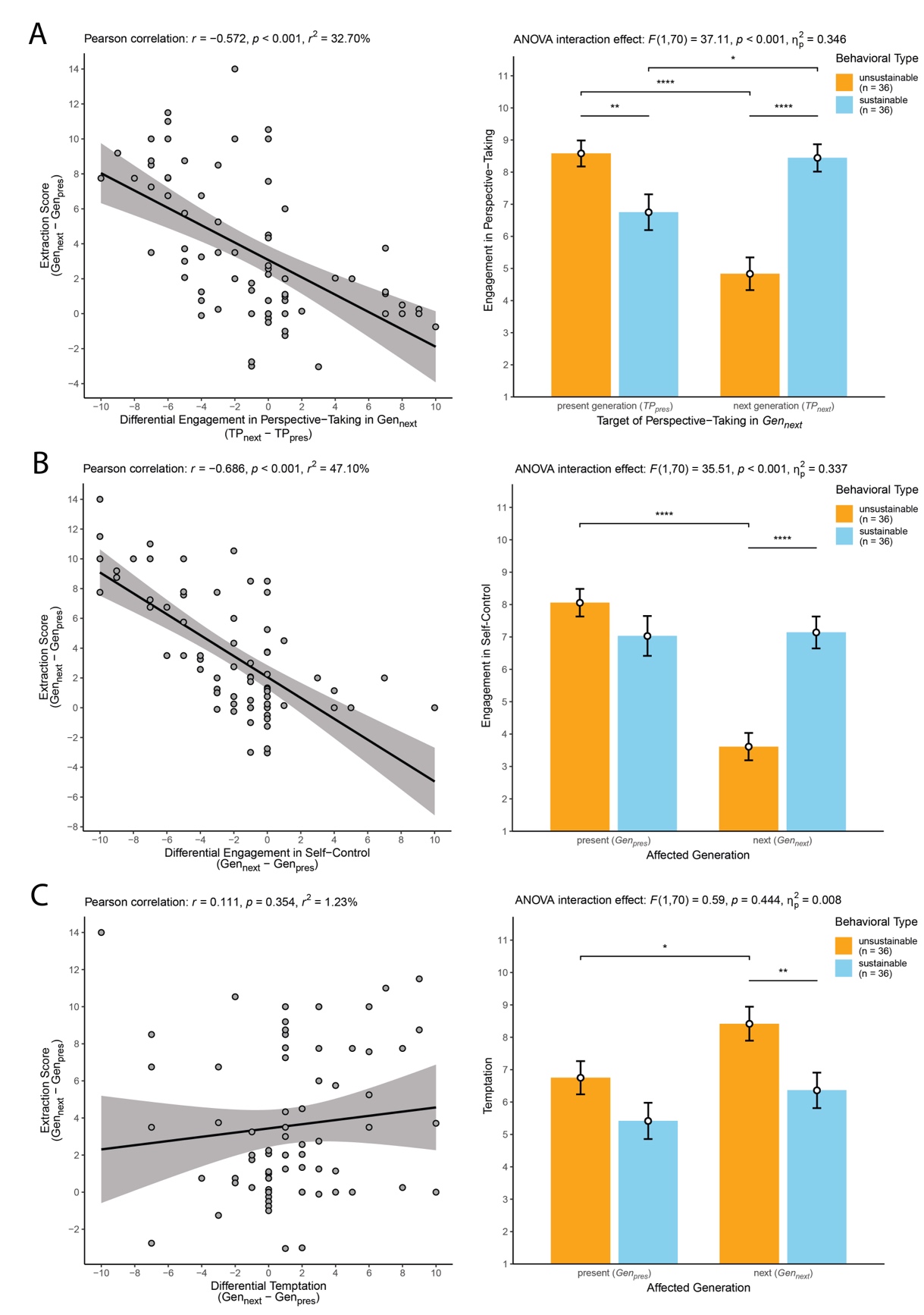
**

***Suppl. Fig. S2.* Subjective ratings of perspective-taking and self-control related processes and their association with intergenerational sustainability.** Depicted in **(A)** is the scatter plot showing the interindividual differences in extraction score (*Gen_next_* – *Gen_pres_*, y-axes) plotted against the interindividual differences in perspective-taking in trials affecting the next generation (*TP_next_* – *TP_pres_*, x-axis). Pearson correlation demonstrated a strong negative association (*r* = -0.572, *p* < 0.001), indicating that the more participants’ perspective-taking was oriented towards the next generation, the more participants behaved sustainably (lower extraction scores). For an improved understanding of the correlational finding, bar plots show the disentangled perspective-taking values, broken down for the two behavioral types (same figure as in the main manuscript, see Fig. 2d). Depicted in **(B)** is the scatter plot showing the interindividual differences in extraction score (*Gen_next_* – *Gen_pres_*, y-axes) plotted against the interindividual differences in engagement in self-control (*Gen_next_* – *Gen_pres_*, x-axes). Pearson correlation demonstrated a strong negative association (*r* = -0.686, p < 0.001), indicating that the more effort participants invested in resisting temptation in *Gen_next_* compared to *Gen_pres_*, the more participants behaved sustainably (lower extraction scores). For an improved understanding of the correlational finding, bar plots show the disentangled values, broken down for the two behavioral types. Bar plots revealed that sustainable participants reported an equal (and rather high) effort to resist temptation in both conditions (*Gen_next_* and *Gen_pres_*), whereas unsustainable participants reported significantly lower efforts to resist temptation in *Gen_next_* trials compared to *Gen_pres_* trials. Depicted in **(C)** is the scatter plot showing the interindividual differences in extraction score (*Gen_next_* -– Gen_pres_, x-axes) plotted against the interindividual differences in temptation (*Gen_next_* – *Gen_pres_*, y-axis). Pearson correlation demonstrated no significant association between behavior and temptation (*r* = 0.111, p = 0.354). Bar plots visualize the disentangled values and demonstrate that both behavioral types reported an average level of temptation in both conditions. However, unsustainable (vs. sustainable) participants reported slightly higher levels of temptation in both conditions (albeit significant only in *Gen_next_*). Regression lines in scatterplots represent lines of best fit surrounded by their corresponding 95% confidence interval. Error bars in bar plots depict standard errors of the means and asterisks denote significant differences (* *p* < 0.05, ** *p* < 0.01, *** *p* < 0.005, **** *p* < 0.001) based on dependent and independent t-tests. For completeness, we also reported the significance of the interaction effects of the two-way mixed ANOVAs with between-subject factor Behavioral Type (unsustainable vs. sustainable) and within-subject factors Affected Generation (*Gen_pres_* vs. *Gen_next_*) or target of perspective-taking (TP_pres_ vs. *TP_pres_*).


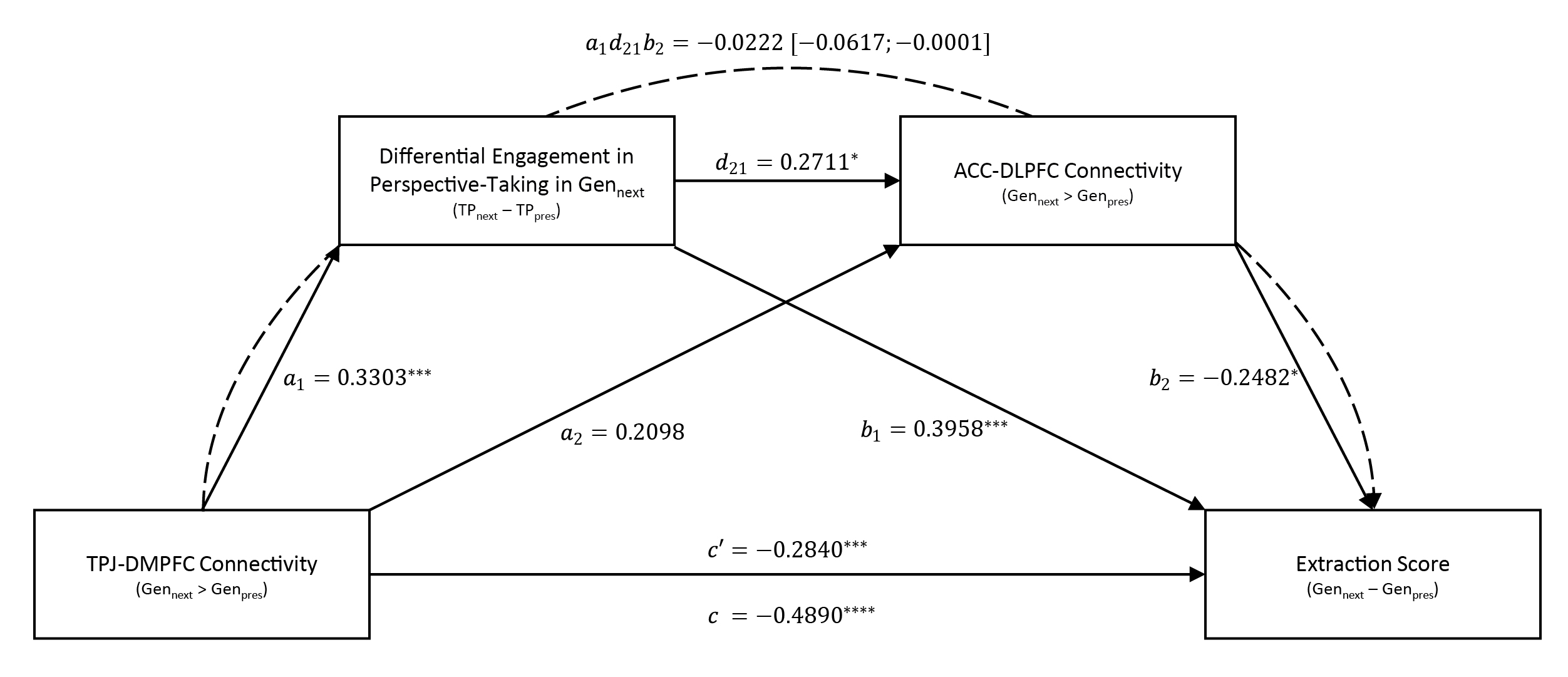


***Suppl. Fig. S3:* Serial mediation analysis investigating a tentative and speculative integrative model with perspective-taking and self-control processes as serial mediators.** Depicted in the path diagram is the serial mediation analysis showing that differential engagement in perspective-taking and ACC-DLPFC connectivity serially mediate the impact of TPJ‑DMPFC connectivity on the extraction score, i.e., stronger functional connectivity between TPJ and DMPFC (*Gen_next_* > *Gen_pres_*) predicted more next-generation oriented perspective-taking (*TP_next_* – *TP_pres_*), which in turn led to a stronger functional connectivity between ACC and DLPFC (*Gen_next_* > *Gen_pres_*), which consecutively was associated with increased intergenerational sustainable behavior. All critical paths and the indirect effect via the two serial mediators were significant. Please note that in the serial mediation model we again controlled for differential temptation, however, the findings hold if we do not control for it. Further, please note that a serial mediation model with an additional third mediator (differential engagement in self-control) does not work anymore due to high correlations between the three mediators, resulting in some paths in the mediation model becoming non-significant. The path diagrams depict standardized linear regression coefficients and asterisks denote significant effects: * p < 0.05, ** p < 0.01, *** p < 0.005, **** p < 0.001. The interval of the indirect effect (*a_1_d_21_b_2_*) represent a 95% confidence interval calculated using 5’000 bootstrap samples (with the value zero not contained in the interval indicating a significant indirect effect). *c* = total effect, *c’* = direct effect.

**Supplementary Analysis**

A multiple regression of the extraction score on altruistic, egoistic, hedonic, and biospheric values assessed by the Schwartz Values Scale (SVS) ^[1]^ revealed that the more individuals endorsed altruistic values, the more sustainably they behaved towards the next generation (see main manuscript). Thus, we aimed for assessing whether the associations between the extraction score and functional activity and connectivity were independent of participants’ altruistic values. To this end, we conducted the same regression analyses as depicted in Fig. 2b and Fig. 3a/b/c by regressing the extraction score on the extracted TPJ activity or TPJ-DMPFC/TPJ-ACC/ACC-DLPFC connectivity but this time entering altruistic values as a covariate of no interest. We found that results held after controlling for altruistic values in the case of TPJ activity (partial Pearson correlation *r_part_* = 0.450, *p* < 0.001, *ΔR^2^* = 18.70%), TPJ‑DMPFC connectivity (*r_part_* = ‑0.4641, *p* < 0.001, *ΔR^2^* = 19.91%), TPJ‑ACC connectivity (*r_part_* = ‑0.421, *p* < 0.001, *ΔR^2^* = 16.36%), and ACC‑DLPFC connectivity (*r_part_* = ‑0.442, *p* < 0.001, *ΔR^2^* = 18.06%). Thus, these neural mechanisms during decision-making uniquely explained variance in intergenerational sustainable behavior over and above participants’ altruistic values.

# Supplementary Discussion

While our connectivity results were mainly driven by sustainable participants’ increased functional connectivity in *Gen_next_* trials, we also observed partly reversed connectivity patterns for unsustainable participants in *Gen_pres_* trials. For these participants, neural communication within and between the mentalizing and cognitive control network tended to be stronger in situations in which overharvesting could reduce their own benefits. While unexpected, this pattern might be tentatively explained by the reasoning that unsustainable participants likely desired to extract as many points as possible to maximize their own benefits. In *Gen_pres_* trials, this might have led to a desire-goal conflict because overharvesting would likely reduce their payoff considerably. Therefore, unsustainable participants might be more prone to monitor and act upon their desire-goal conflicts in *Gen_pres_* trials by deploying increased self-control processes to resist the temptation of overharvesting (TPJ-ACC and ACC-DLPFC connectivity). This reasoning resonates with accumulating evidence in single-generation contexts showing that self-control processes depend on individual and situational characteristics ^[2–11]^.

In addition to functional connectivity within and between the hypothesized mentalizing and cognitive control networks, we also found that increased TPJ‑aINS and TPJ‑HC connectivity in *Gen_next_* (vs. *Gen_pres_*) trials was associated with more sustainable behavior (see Suppl. Fig. S1). While these findings were beyond the scope of our hypotheses, we provide some tentative interpretations for the sake of completeness.

The aINS is known to be involved in affective responses, including empathy ^[12,13]^. For instance, a deficit of empathy related aINS activity when processing information pertaining to outgroup compared to ingroup members has been suggested to underly single-generation intergroup bias ^[14–17]^. Therefore, one might speculate that greater TPJ‑aINS connectivity represents greater engagement in empathic processes targeting future others, in parallel to future generation oriented perspective-taking processes supported by TPJ-DMPFC connectivity. This aligns with accounts suggesting dissociable and shared neural networks supporting empathy and perspective-taking, which regard the former as a more affective and the latter as a more cognitive form of putting oneself in others’ shoes ^[18–20]^.

The HC is known to be crucially involved not only in forming and recalling episodic memories but also in mentally simulating future events, which has been argued to play an important role in deliberating over intertemporal trade-offs ^[21,22]^. More specifically, a recent study found that HC activity was related to prospective thinking about daily pro-environmental behavior, possibly by supporting the simulation of sustainable behavior based on episodic memories ^[23]^. In line with recent theoretical considerations attributing a role of prospective thinking and its neural implementation for sustainable behavior ^[24]^, increased TPJ-HC connectivity might enable individuals to more vividly simulate prospective events affecting future others, aiding intergenerational perspective-taking and ultimately promoting sustainable behavior. Future studies more closely focusing on these additional potential processes (affective processes/empathy and prospective thinking) could broaden our understanding of the neural processes driving interindividual differences in intergenerational sustainable behavior.

# References

1. Steg, L., Perlaviciute, G., van der Werff, E. & Lurvink, J. The Significance of Hedonic Values for Environmentally Relevant Attitudes, Preferences, and Actions. *Environ. Behav.* **46**, 163–192 (2014).

2. Gianotti, L. R. R., Nash, K., Baumgartner, T., Dahinden, F. M. & Knoch, D. Neural signatures of different behavioral types in fairness norm compliance. *Sci. Rep.* **8**, 10513 (2018).

3. Hackel, L. M., Wills, J. A. & Van Bavel, J. J. Shifting prosocial intuitions: neurocognitive evidence for a value-based account of group-based cooperation. *Soc. Cogn. Affect. Neurosci.* **15**, 371–381 (2020).

4. Hampton, A. N., Bossaerts, P. & O’Doherty, J. P. Neural correlates of mentalizing-related computations during strategic interactions in humans. *PNAS* **105**, 6741–6746 (2008).

5. Hill, C. A. *et al.* A causal account of the brain network computations underlying strategic social behavior. *Nat. Neurosci.* **20**, 1142–1149 (2017).

6. Pierce, J. R., Kilduff, G. J., Galinsky, A. D. & Sivanathan, N. From Glue to Gasoline: How Competition Turns Perspective Takers Unethical. *Psychol. Sci.* **24**, 1986–1994 (2013).

7. Sassenrath, C., Vorauer, J. D. & Hodges, S. D. The link between perspective-taking and prosociality — Not as universal as you might think. *Curr. Opin. Psychol.* **44**, 94–99 (2022).

8. Speer, S. P. H., Smidts, A. & Boksem, M. A. S. Cognitive control and dishonesty. *Trends Cogn. Sci.* **26**, 796–808 (2022).

9. Spitzer, M., Fischbacher, U., Herrnberger, B., Grön, G. & Fehr, E. The Neural Signature of Social Norm Compliance. *Neuron* **56**, 185–196 (2007).

10. Wyss, A. M. & Knoch, D. Neuroscientific approaches to study prosociality. *Curr. Opin. Psychol.* **44**, 38–43 (2022).

11. Yamagishi, T. *et al.* Cortical thickness of the dorsolateral prefrontal cortex predicts strategic choices in economic games. *Proceedings of the National Academy of Sciences* **113**, 5582–5587 (2016).

12. Lamm, C. & Singer, T. The role of anterior insular cortex in social emotions. *Brain Struct. Funct.* **214**, 579–591 (2010).

13. Rilling, J. K. & Sanfey, A. G. The Neuroscience of Social Decision-Making. *Annu. Rev. Psychol.* **62**, 23–48 (2011).

14. Azevedo, R. T. *et al.* Their pain is not our pain: Brain and autonomic correlates of empathic resonance with the pain of same and different race individuals. *Hum. Brain Mapp.* **34**, 3168–3181 (2013).

15. Contreras-Huerta, L. S., Baker, K. S., Reynolds, K. J., Batalha, L. & Cunnington, R. Racial Bias in Neural Empathic Responses to Pain. *PLoS ONE* **8**, e84001 (2013).

16. Hein, G., Silani, G., Preuschoff, K., Batson, C. D. & Singer, T. Neural responses to ingroup and outgroup members’ suffering predict individual differences in costly helping. *Neuron* **68**, 149–160 (2010).

17. Hein, G., Morishima, Y., Leiberg, S., Sul, S. & Fehr, E. The brain’s functional network architecture reveals human motives. *Science* **351**, 1074–1078 (2016).

18. Healey, M. L. & Grossman, M. Cognitive and Affective Perspective-Taking: Evidence for Shared and Dissociable Anatomical Substrates. *Frontiers in Neurology* **9**, 491 (2018).

19. Schurz, M. *et al.* Toward a hierarchical model of social cognition: A neuroimaging meta-analysis and integrative review of empathy and theory of mind. *Psychol. Bull.* **147**, 293–327 (2021).

20. Stietz, J., Jauk, E., Krach, S. & Kanske, P. Dissociating Empathy From Perspective-Taking: Evidence From Intra- and Inter-Individual Differences Research. *Front. Psychiatry* **10**, (2019).

21. Bulley, A. & Schacter, D. L. Deliberating trade-offs with the future. *Nat. Hum. Behav.* **4**, 238–247 (2020).

22. Schacter, D. L. *et al.* The Future of Memory: Remembering, Imagining, and the Brain. *Neuron* **76**, 677–694 (2012).

23. Brevers, D. *et al.* Brain mechanisms underlying prospective thinking of sustainable behaviours. *Nat. Sustain.* **4**, 433–439 (2021).

24. Aoki, R., Ito, A., Izuma, K. & Saijo, T. How can neuroscience contribute to the science of intergenerational sustainability? (2020).
